# The Ethics of Using Urotropine

**Published:** 1901-08-24

**Authors:** 


### THE ETHICS OF USING UROTROPINE.

The above suggestion that, with the object of destroying the typhoid bacilli so often present in the urine of patients suffering from enteric fever, all such patients should undergo a course of urotropine, raises certain ethical questions. The observation that urotropine occasionally produces hæmaturia, has led Dr. Handford, of Nottingham, to say that however advantageous it may be from a public health point of view to kill off the bacilli in this way, the question arises whether it is justifiable to administer a drug to a patient with any other object than his own cure, especially when it has been shown that the drug in question may, and does occasionally, do harm. We agree with him in his protest against the routine use of this drug for any such purpose as disinfecting the urine for the public good. Any drug which might give rise to hæmaturia ought only to be given when of benefit to the patient. We must find other means for rendering the urine innocuous as we have perforce to do in the case of the other excreta.

